# Molecular Investigation of Mitochondrial RNA19 Role in the Pathogenesis of MELAS Disease

**DOI:** 10.3390/life13091863

**Published:** 2023-09-03

**Authors:** Paola Loguercio Polosa, Francesco Capriglia, Francesco Bruni

**Affiliations:** Department of Biosciences, Biotechnologies and Environment, University of Bari ‘Aldo Moro’, 70125 Bari, Italy; paolaannamaria.loguerciopolosa@uniba.it (P.L.P.); f.capriglia@sheffield.ac.uk (F.C.)

**Keywords:** mt-RNA processing, RNA19, MELAS, trans-mitochondrial cybrids, mitochondrial translation, mitoribosome, LARS2, Cterm, mitochondrial disease

## Abstract

In mammalian mitochondria, the processing of primary RNA transcripts involves a coordinated series of cleavage and modification events, leading to the formation of processing intermediates and mature mt-RNAs. RNA19 is an unusually stable unprocessed precursor, physiologically polyadenylated, which includes the 16S mt-rRNA, the mt-tRNALeu^UUR^ and the mt-ND1 mRNA. These peculiarities, together with the alteration of its steady-state levels in cellular models with defects in mitochondrial function, make RNA19 a potentially important molecule for the physiological regulation of mitochondrial molecular processes as well as for the pathogenesis of mitochondrial diseases. In this work, we quantitatively and qualitatively examined RNA19 in MELAS trans-mitochondrial cybrids carrying the mtDNA 3243A>G transition and displaying a profound mitochondrial translation defect. Through a combination of isokinetic sucrose gradient and RT-qPCR experiments, we found that RNA19 accumulated and co-sedimented with the mitoribosomal large subunit (mt-LSU) in mutant cells. Intriguingly, exogenous expression of the isolated LARS2 C-terminal domain (Cterm), which was shown to rescue defective translation in MELAS cybrids, decreased the levels of mt-LSU-associated RNA19 by relegating it to the pool of free unbound RNAs. Overall, the data reported here support a regulatory role for RNA19 in mitochondrial physiopathological processes, designating this RNA precursor as a possible molecular target in view of therapeutic strategy development.

## 1. Introduction

The human mitochondrial genome (mtDNA) consists of a circular molecule, 16,569 bp long, encompassing thirty-seven genes that encode 2 mt-rRNAs, 22 mt-tRNAs and 11 mt-mRNAs. Mitochondrial RNAs (mt-RNAs) are required for the synthesis of thirteen polypeptides that assemble with nucleus-encoded mitochondrial proteins to form respiratory chain complexes [1]. Mt-RNAs are first synthesised as extended polycistronic transcripts. Subsequently, in order to be functional for protein synthesis, these long precursors undergo maturation by coordinated processes of cleavage and post-transcriptional modification, both taking place within the mitochondrial RNA granules [2,3]. The enzymatic cleavage at the 5′ and 3′ ends of mt-tRNAs is mediated by RNase P and ELAC2, respectively, and is crucial for releasing mature mt-RNAs co-transcriptionally or shortly after RNA synthesis [4]. However, mt-tRNA excision is not 100% efficient in all sites, and several persistent processing intermediates are experimentally detectable [5]. The enzymatic cleavage efficiency can be either tissue-specific or influenced by mutations that alter the processed sequences [6].

One of the most stable and well-characterised mitochondrial precursors is RNA19, a 2.6 kb long unprocessed intermediate consisting of the linked 16S mt-rRNA, mt-tRNALeu^UUR^ and mt-mRNA encoding ND1, whose respective genes are adjacent on the mtDNA. This transcript was identified for the first time about thirty years ago from a cellular system of trans-mitochondrial cybrids used as a model to study the pathological effects of the 3243A>G mutation, placed within the loop D of mt-tRNALeu^UUR^ and most frequently reported in patients with MELAS (Mitochondrial Encephalopathy with Lactic Acidosis and Stroke-like Episodes) syndrome [7,8,9]. Notably, RNA19 was also detectable in wild-type cybrids, but a significant increase in steady-state levels was observed in mutant cells. The fact that RNA19 has also been identified in non-mutant cells certainly has physiological relevance; however, most studies have been investigating the detrimental effect of point mutations on the processing of this specific RNA precursor. Employing an in vitro tRNA processing system that faithfully reflected the in vivo cleavage mediated by RNase P and ELAC2, researchers demonstrated the correlation between different mutations lying in the mt-tRNALeu^UUR^ and the accumulation of RNA19 [10,11]. Several other studies on either MELAS syndrome or other mitochondrial disorders reported augmented steady-state levels of RNA precursors, particularly RNA19 [6,12,13,14,15,16].

Recent research from our laboratory showed that RNA19 can in cellulo bind the carboxy-terminal domain of the human mitochondrial leucyl-tRNA synthetase, LARS2 [17]. Overexpression of this isolated domain, concisely named Cterm, has proven effective in rescuing the defective phenotype associated with the 3243A>G transition in human cells [18,19]. Importantly, exogenous expression of the Cterm was able to recover de novo mitochondrial protein synthesis, ameliorating the viability of MELAS cybrids [17]. However, to date, the molecular mechanisms of Cterm-mediated rescue are still unknown.

Here, we decided to deepen our knowledge of the processes underlying the Cterm biological activity. Firstly, the role of RNA19 in MELAS cybrids was investigated, exploring its possible association with mitoribosomes. Further, the interaction of the Cterm peptide with RNA19 and its potential effect on defective mitochondrial translation were examined.

## 2. Materials and Methods

### 2.1. Tissue Culture and Heteroplasmy Determination

Trans-mitochondrial cybrids and HEK293T cells were cultured at 37 °C and humidified 5% CO_2_ in Dulbecco’s modified Eagle’s medium (DMEM; Lonza, Basel, Switzerland), supplemented with 10% (*v*/*v*) Foetal Bovine Serum (FBS; ThermoFisher Scientific, Carlsbad, CA, USA), 1 mM sodium pyruvate, 1× non-essential amino acids, 50 µg/mL uridine and 1× Antibiotic-Antimycotic (Euroclone, Milan, Italy).

For PCR-RFLP determination of the 3243A>G mutation load, total DNA was extracted from cybrids using the Wizard^®^ Genomic DNA Purification Kit (Promega, Madison, WI, USA), following manufacturer’s recommendations. The 3243 locus on the human mitochondrial genome was amplified using the following primers: Melas.FOR, 5′-CCTCGGAGCAGAACCCAACCT-3′; and Melas.REV, 5′-CGAAGGGTTGTAGTAGCCCGT-3′. After PCR reactions, the amplified products were ApaI-digested, fractionated on 1.5% agarose and stained with GelRed^®^ (Biotium, Fremont, CA, USA). The proportion of mutant (digested) to wild-type (undigested) mtDNA fragments was determined by ImageQuant TL 8.1 software (GE Healthcare Life Sciences, Pittsburgh, PA, USA).

### 2.2. Construct Preparation and Cterm Expression

The construct to facilitate inducible expression of the isolated carboxy-terminal domain of LARS2 (Cterm), fused with Cox8 mitochondrial presequence at the N-terminus and FLAG-tag at the C-terminus, was prepared from the plasmid pcDNA6.2/Cox8-Cterm-FLAG [17]. The plasmid was NotI-/ApaI-digested, and the obtained insert was gel-eluted and ligated into the tetracycline-inducible pcDNA5/FRT/TO^®^ vector (Invitrogen, Waltham, MA, USA), according to standard cloning techniques. Cterm construct was finally validated by sequencing both DNA strands. The empty pcDNA5/FRT/TO^®^ vector was used as mock control plasmid for transfections performed in MELAS cybrids. Transfections were carried out with Lipofectamine™ 3000 Transfection Reagent (ThermoFisher Scientific, Carlsbad, CA, USA), following manufacturer’s recommendations.

The day following transfection, Cterm expression was induced by growing the cultures in the presence of 1 μg/mL tetracycline. After 48 h, whole-cell or mitochondrial extracts were prepared, and Cterm expression and localization were assessed using immunoblotting (as described in Section 2.5). Whole-cell extracts were obtained by solubilization in lysis buffer (50 mM Tris-HCl, pH 7.4; 150 mM NaCl; 1 mM EDTA; 1% Triton X-100; cOmplete™ Protease Inhibitor Cocktail (Roche, Basel, Switzerland)), followed by incubation at 4 °C for 30 min. Extracts were cleared by 15 min centrifugation at 12,000× *g.*

For the localization experiment, mitochondrial lysates were prepared from HEK293 cells transfected with the Cterm plasmid, following the procedure described in [20] with few modifications. Briefly, Cterm-expressing cells were harvested, resuspended in H-buffer (0.6 M mannitol; 10 mM Tris-HCl, pH 7.4; 1 mM EGTA), supplemented with 0.1% BSA (*w*/*v*) and subjected to standard differential centrifugation. Mitochondria were pelleted (11,000× *g*, 10 min, 4 °C) and resuspended in H-buffer. The post-mitochondrial supernatant (cytosolic fraction) was retained after centrifugation. Intact mitochondria were treated with proteinase K (5 ng/µg mitochondrial proteins) on ice for 30 min, followed by addition of 5 mM PMSF, pelleted, and resuspended in H-buffer. After preparation, both whole-cell and mitochondrial lysates were snap-frozen and stored at −80 °C.

### 2.3. Immunoprecipitation of Cterm-FLAG

MELAS cybrids, transfected stably with either the Cterm-FLAG plasmid or the empty vector (mock), were grown at high confluency in 2 × 500 cm^2^ square dishes (Corning, Corning, NY, USA), washed with prechilled PBS, crosslinked in 1% formaldehyde–PBS solution for 10 min at room temperature and treated for 5 min with 0.125 M glycine, pH 7.0. Mitochondria were prepared as described in Section 2.2, and pellets were dissolved in low-salt buffer (50 mM Tris-HCl, pH 7.4; 150 mM NaCl; 0.05% IGEPAL), freshly added with 1× Protease Inhibitor Cocktail (Cell Signaling Technology, Danvers, MA, USA). To isolate the mitochondrial soluble proteins, samples were rotated for 30 min in a cold room and centrifuged at 10,000× *g* for 10 min at 4 °C. The soluble fractions (600 μg) were immunoprecipitated with anti-FLAG^®^ M2 affinity gel (Sigma Aldrich, St. Louis, MO, USA), as described in [21]. After crosslink reversion, immunoprecipitated Cterm-FLAG peptide was detected using Western blotting (see Section 2.5).

### 2.4. Isokinetic Sucrose Gradient Analysis

Whole-cell lysates (1 mg) were prepared as described above in lysis buffer, freshly complemented with 10 mM MgCl_2_, 1 mM AEBSF and 0.5 U/µL RiboLock RNase Inhibitor (ThermoFisher Scientific, Carlsbad, CA, USA), and loaded onto a 10–30% (*v*/*v*) sucrose gradient in 50 mM Tris-HCl, pH 7.2, 40 mM NH_4_Cl, 100 mM KCl, 10 mM MgOAc, 1 mM PMSF and 50 µg/mL chloramphenicol. Gradients were subjected to centrifugation in an Optima L-100K ultracentrifuge (Beckman Coulter, Brea, CA, USA), using a SW 40Ti rotor at 130,000× *g* for 2 h and 15 min at 4 °C. Eleven fractions were collected and analysed using immunoblotting or, following RNA extraction, RT-qPCR.

### 2.5. Western Blotting

Variable amounts (depending on the proteins being probed) of whole-cell proteins, mitochondrial lysates or sucrose gradient fractions were solubilised in 1× Laemmli buffer and fractionated onto 12% Tris-Glycine-SDS minigels. Proteins from gels were electro-transferred onto polyvinylidene difluoride (PVDF) membranes (Millipore, Burlington, MA, USA) at 300 mA for 1 h and 30 min at 4 °C. The immunoblotting procedure was executed according to standard protocol. Primary antibodies (all diluted 1:1000, except where differently indicated) were as follows: total OXPHOS antibody cocktail (ab110413, 1:250 dilution; Abcam, Cambridge, UK), anti-COXI (ab14705; Abcam, Cambridge, UK), anti-mS29 (MA141279; Life Technologies, Carlsbad, CA, USA), anti-uL13m (16241-1-AP; ProteinTech, Manchester, UK), anti-mL37 (HPA025826; Atlas Antibodies AB, Bromma, Sweden), anti-FLAG (F1804; Sigma Aldrich, St. Louis, MO, USA), anti-GSR (LF-PA0056; ThermoFisher Scientific, Carlsbad, CA, USA) and anti-VDAC1 (ab186321; Abcam, Cambridge, UK). Following incubation with horseradish peroxidase-conjugated secondary antibodies (swine anti-rabbit IgG, P039901-2; rabbit anti-mouse IgG, P026002-2; 1:2500 dilution; Agilent Dako, Santa Clara, CA, USA), detection was performed using Clarity Western ECL substrate (Bio-Rad, Hercules, CA, USA), and chemiluminescent signals were revealed by the ChemiDoc MP Imaging System (Bio-Rad, Hercules, CA, USA).

### 2.6. RT-qPCR Analysis

RNA samples, prepared from either whole cells or gradient fractions by TRIsure™ reagent (Meridian Bioscience, Cincinnati, OH, USA), were reverse-transcribed using the High-Capacity cDNA Reverse Transcription Kit (ThermoFisher Scientific, Carlsbad, CA, USA), according to the manufacturer’s guidelines. For RNA19 transcript analysis, primers were as follows: 16S.FOR, 5′-TATACCCACACCCACCCAAG-3′; and ND1.REV, 5′-GCGATTAGAATGGGTACAAT-3′. Cterm RNA assessment was carried out with the following primers: Cterm.FOR, 5′-AAATTCCTGTGCCCCAACAA-3′; and FLAG.REV, 5′-CTACTTATCGTCGTCATCCT-3′. The reference transcript was amplified with hs18S.FOR, 5′-GTAACCCGTTGAACCCCATT-3′; and hs18S.REV, 5′-TGAAGAACAAATTCGTGGACTTTG-3′.

Amplification reactions were carried out using the SsoAdvanced Universal SYBR^®^ Green Supermix (BioRad, Hercules, CA, USA) and analysed with the Applied Biosystem 7500 Fast Real-Time PCR System (ThermoFisher Scientific, Carlsbad, CA, USA). Relative quantification of the qPCR products was achieved using the Pfaffl method [22]; statistical analysis was performed using the unpaired two-tailed Student’s *t* test.

### 2.7. Pulse-Labelling of Mitochondrial Translation Products

De novo mitochondrial protein synthesis was assessed on exponentially growing cybrids in 12-well plates. Cells were washed twice in methionine-free DMEM (Sigma, St. Louis, MO, USA) and pulsed with 300 μCi/mL [^35^S]-methionine (PerkinElmer, Waltham, MA, USA) at 37 °C for 1 h in 300 μL of methionine-free DMEM supplemented with 10% dialysed FBS, emetine (100 µg/mL) and cycloheximide (100 µg/mL). After the radioactive pulse, cells were washed twice with phosphate-buffered saline (PBS) and dissolved in 1× Laemmli buffer. Aliquots (50 μg) of total cell protein were fractionated by 15% SDS-PAGE and radioactive signals were detected using the Typhoon FLA 9500 PhosphorImager and ImageQuant TL 8.1 software (GE Healthcare Life Sciences, Pittsburgh, PA, USA). Post-detection protein loading was determined using Coomassie blue staining.

## 3. Results

### 3.1. Mitoribosomal Subunits Were Normally Assembled in Translation-Defective MELAS Cybrids

**Figure 1:**
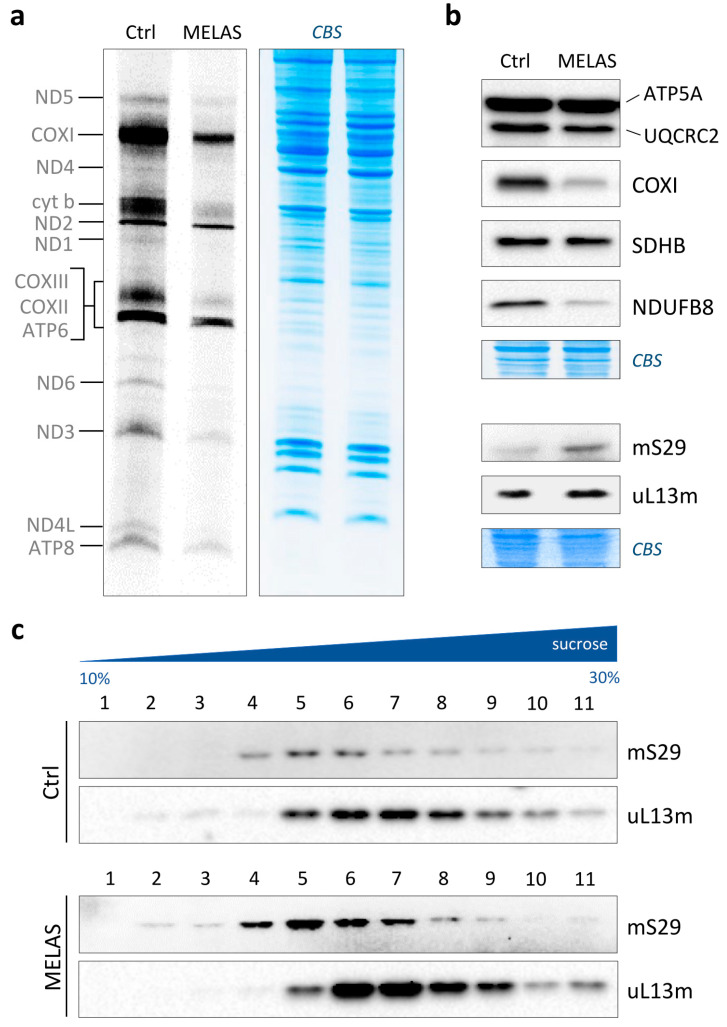
Mitochondrial translation in MELAS cybrids. (**a**) Metabolic [^35^S]-methionine labelling of de novo synthesised mitochondrial polypeptides in wild-type (Ctrl) and MELAS cybrids. Pulse-labelling was carried out for 1 h at 37 °C; total cell proteins (50 µg) were separated onto 15% SDS-PAA gels. On the left, mitochondrially encoded polypeptides are denoted. Coomassie blue staining (CBS) of the gel was used as loading control. (**b**) Whole-cell lysates were prepared, and aliquots (40 µg) subjected to immunoblotting (as detailed in Section 2.5). Steady-state levels of representative subunits of Complex I (NDUFB8), Complex II (SDHB), Complex III (UQCRC2), Complex IV (COXI) and Complex V (ATP5A) were examined using OXPHOS antibody cocktail. Steady-state levels of mitoribosomal components were determined using antibodies against marker proteins of the mt-SSU (mS29; HUGO protein name: DAP3) and the mt-LSU (uL13m; HUGO protein name: MRPL13). Coomassie blue staining (CBS) of the membranes was used as transfer control. (**c**) Analysis of mitoribosomal protein distribution in wild-type (Ctrl) and MELAS cybrids through isokinetic sucrose gradient centrifugation. The migration of the mt-SSU and mt-LSU was determined using antibodies as described in (**b**).

We employed a MELAS cybrid trans-mitochondrial cybrid line carrying the 3243A>G transition within the MT-TL1 gene encoding mt-tRNALeu^UUR^. First, the heteroplasmy level of the mutation was determined using PCR-RFLP analysis. We measured a >85% mutation load in MELAS compared to wild-type cybrids (Figure S1). To examine the de novo synthesis of mitochondrial polypeptides in these cell lines, metabolic labelling was performed in the presence of [^35^S]-methionine and inhibitors of cytosolic translation. MELAS cells showed considerably reduced levels of mitochondrial protein synthesis (Figure 1a); the decrease extended to all thirteen mitochondria-encoded polypeptides.

It is known that not all lines carrying the 3243A>G mutation show quantitative defects in mitochondrial protein synthesis. However, a common molecular phenotype in these cell types is certainly a deficiency of respiratory Complexes I and IV. To confirm this phenotype in our cell line, we measured the steady-state levels of five representative polypeptides of each OXPHOS complex using immunoblotting. We observed a strong decrease in the NDUFB8 (Complex I) and COXI (Complex IV) subunits in MELAS cells with respect to the control, without any substantial variation for the other polypeptides under examination (Figure 1b, upper panel). The same analysis was also performed on mS29 and uL13m, two mitoribosomal proteins belonging to the mt-SSU and mt-LSU, respectively. The steady-state levels of both MRPs were increased in MELAS cybrids, with mS29 at a higher extent (Figure 1b, lower panel), likely to compensate for defective protein synthesis.

The observed profound translation defect, together with the fact that the faulty production of mitoribosomes significantly contributes to the development of metabolic and neurodegenerative diseases [23], prompted us to test the assembly of ribosomal subunits in both the control and MELAS cybrids. The profile of the mitoribosomes was examined on an isokinetic sucrose gradient, and the analyses with the marker proteins mS29 and uL13m showed that both the mt-SSU and mt-LSU, respectively, were correctly assembled in the MELAS cybrids, with no change in their pattern compared to the control cells (Figure 1c). Therefore, the high levels of mutation load had heavy effects on mitochondrial translation without negatively altering mitoribosome assembly.

### 3.2. RNA19 Accumulated in the Gradient Denser Fractions from Mutant Cells

We previously demonstrated that the Cterm peptide can bind RNA19 [17]. It has been observed that this transcript accumulates in the presence of several pathogenic mutations associated with MELAS syndrome, particularly the 3243A>G transition. On this basis, we focused our analysis on RNA19 in our MELAS cell line.

**Figure 2:**
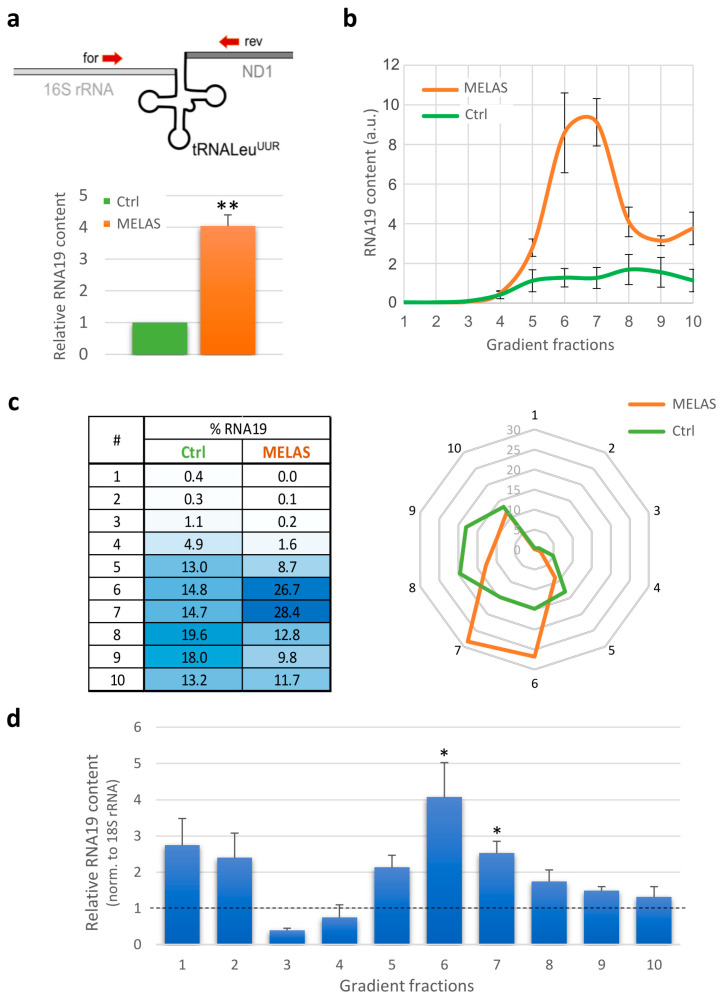
Quantitative and qualitative analyses of RNA19 transcript. (**a**) Relative quantification of RNA19 steady-state levels using RT-qPCR. Upper: Schematic diagram of the precursor encompassing 16S mt-rRNA (light grey line), mt-tRNALeu^UUR^ (black ‘cloverleaf’) and mt-ND1 mRNA (dark grey line). Specific forward and reverse primers (red arrows) were designed upstream and downstream of the tRNA sequence, respectively. Lower: The RNA19 content in MELAS cells (orange) was compared to wild-type (Ctrl) cybrids (green, fixed as 1-value), normalised to 18S rRNA (reference transcript). Results are presented as the mean ± SE (*n* = 3). Statistical analysis was performed using the two-tailed Student’s *t* test (**, *p* < 0.01). (**b**) The RNA19 abundance in sucrose gradient fractions from control (green) and MELAS (orange) cybrids was determined using RT-qPCR analysis. The data are expressed as arbitrary units, and representative curves are presented as the mean ± SE (*n* = 3). (**c**) The distribution of RNA19 in the different fractions was determined as $2^{-C_{t,f}}/\sum_{f=1}^{10}2^{-C_{t,f}}\times 100$, where C_t_ represents the threshold cycle and f denotes the fraction number. The data are graphically represented by a heat table (left) and a radar chart (right). (**d**) Bars represent the relative RNA19 content for each gradient fraction calculated as the ratio between MELAS and wild-type (Ctrl) cybrids (dotted line, fixed as 1-value), normalised to 18S rRNA. Results are presented as the mean ± SE (*n* = 3). Statistical analysis was performed using the two-tailed Student’s *t* test (*, *p* < 0.05).

Firstly, we carried out a relative quantification of its steady-state levels using RT-qPCR. To this end, we used forward and reverse primers that annealed externally to mt-tRNALeu^UUR^ on the 16S mt-rRNA and mt-ND1 sequences (Figure 2a, upper panel). In this way, we were able to specifically detect RNA19 avoiding the amplification of mature 16S mt-rRNA, mt-tRNALeu^UUR^ and mt-ND1 transcripts. The MELAS cybrids showed an approximately four-fold increase in RNA19 compared to the control (Figure 2a, lower panel), consistent with the findings previously reported for different mitochondrial pathogenic models [6,12,13,14,15,16,24,25,26,27].

This result, together with the evidence that the mutant cells displayed a large deficit in mitochondrial translation, prompted us to verify whether RNA19 was associated with the mitoribosomes. To this purpose, total protein lysates obtained from the control and MELAS cells were subjected to an isokinetic sucrose gradient, and RNA was extracted from each fraction. As shown in Figure 2b, RNA19 was more abundant in the denser fractions in both cell types; interestingly, fractions 6 and 7 of the MELAS cells were significantly enriched compared to the control cybrids. The same pattern was observed in wild-type HEK293 cells (Figure S2), suggesting that most of the RNA19 is physiologically part of larger molecular complexes. To analyse the distribution of RNA19 in the various fractions of the gradient, the percentage was measured as the ratio between its content in each fraction and the total signal. Fractions 6 and 7 in the MELAS cells contained more than 50% of the total RNA19 content, whereas in the control cybrids, this RNA was more equally distributed along the gradient denser fractions (Figure 2c).

To assess the change in the RNA19 amount for each fraction, relative quantification was performed in the MELAS cells compared to the control cells, using 18S rRNA as a reference transcript. Following reverse transcription, qPCR results confirmed a significant increase in fractions mainly containing the large subunit of the mitoribosome (Figure 2d). Hence, RNA19 levels increased in cells with the 3243A>G mutation and co-sedimented with the mt-LSU through gradient sedimentation experiments.

### 3.3. Cterm Did Not Associate with Mitoribosomal Subunits

The observation that the Cterm peptide can bind RNA19 and the circumstance that most of the RNA19 co-sedimented with the mt-LSU under pathological conditions led us to test the possible association between the Cterm and the mitoribosomes. This, in turn, would shed light on the functional significance of the interaction between the Cterm and RNA19.

**Figure 3:**
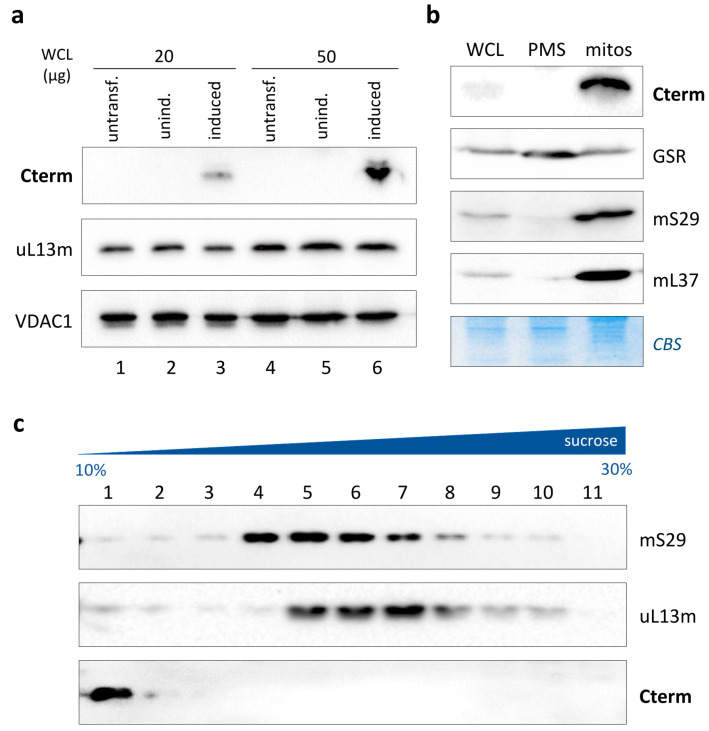
Inducible expression of isolated LARS2 C-terminal domain. (**a**) Western blots of whole-cell lysates (20 µg and 50 µg) from untransfected, uninduced and induced HEK293 cells were probed with antibodies against the FLAG epitope (Cterm), uL13m and VDAC1, the latter being used as a loading control. (**b**) Mitochondrial localisation of Cterm was determined using immunoblotting. Aliquots (40 µg) of whole-cell lysate (WCL), post-mitochondrial fraction (PMS) and mitochondria (mitos) from Cterm-expressing HEK293 were subjected to Western blotting with antibodies against the FLAG epitope (Cterm) and marker proteins of the cytosolic/mitochondrial compartments (GSR, glutathione reductase) and mitochondrial matrix (mS29 and mL37). Coomassie blue staining (CBS) of the membranes was used as loading and transfer control. (**c**) Analysis of Cterm distribution through isokinetic sucrose gradient centrifugation. Gradient fractions were subjected to Western blotting with antibodies against the FLAG epitope (Cterm), mS29 (mt-SSU) and uL13m (mt-LSU).

To this aim, we set out to express the peptide at detectable levels by transiently transfecting a human HEK293T cell line with a pcDNA5-based construct that inducibly expressed the Cterm carrying a C-terminal FLAG. After induction, cells were fractionated and analysed by immunoblotting using an anti-FLAG antibody (Figure 3a). A clear product of the expected length (approx. 12 kDa) was observed in the induced cells (lanes 3 and 6), while no signal was detectable in the untransfected (lanes 1 and 4) or uninduced transfected (lanes 2 and 5) cells. The Cterm was expressed very efficiently in these cells since it was also detectable in a low amount of total protein (20 μg). The peptide was fused in-frame with the N-terminal mitochondrial presequence Cox8, widely used to target proteins to the mitochondrial matrix with high competence. To assess whether the mitochondria-targeted Cterm was successfully imported, mitochondria were isolated, and total protein, post-mitochondrial supernatant (containing cytosolic fraction) and mitochondrial lysate were subjected to Western blotting. As shown in Figure 3b, the Cterm was undetectable in the cytosolic fraction, whereas it displayed a clear co-localization with mitoribosomal proteins mS29 and mL37, used as mitochondrial markers, indicating an elevated level of mitochondrial import of the expressed product. This result is consistent with previous Cterm localization data obtained using either biochemical import and immunofluorescence experiments [18] or subcellular fractionation [19].

In this experimental setting, which ensured high mitochondrial levels of Cterm, we decided to assess the possible interaction of the peptide with the mitoribosomes, specifically with the mt-LSU. To this purpose, a mitochondrial lysate from Cterm-expressing HEK293 cells was fractionated through a 10–30% isokinetic sucrose density gradient, and proteins were separated as detailed. The immunoblotting analysis of the obtained fractions showed that the Cterm possessed a sedimentation profile different from that of mS29 and uL13m, marker proteins for the mt-SSU and mt-LSU, respectively (Figure 3c). Notably, an intense band was detected in fraction 1 by the anti-FLAG antibody, followed by nearly undetectable products in subsequent fractions. These results indicated that the exogenously expressed LARS2 C-terminal peptide did not associate with the ribosomal subunits. Instead, it sedimented in the top gradient fraction, consisting of low-molecular-weight components such as pools of tRNA (Figure S3) and proteins that are not involved in the formation of larger complexes [28].

In light of the Cterm/RNA19 interaction previously demonstrated by us [17], overall, these data suggest that the Cterm would interact preferentially with the free pool of both mt-tRNALeu^UUR^ and its precursor RNA19.

### 3.4. Cterm Expression Affected RNA19 Sedimentation Profile

Our findings showed that, under pathological conditions, more than half of the RNA19 content sedimented in mt-LSU-containing fractions (Figure 2). To investigate the effect of the Cterm peptide on the steady-state level of RNA19 as well as its sedimentation profile, we analysed this transcript in MELAS cybrids overexpressing the Cterm peptide.

**Figure 4:**
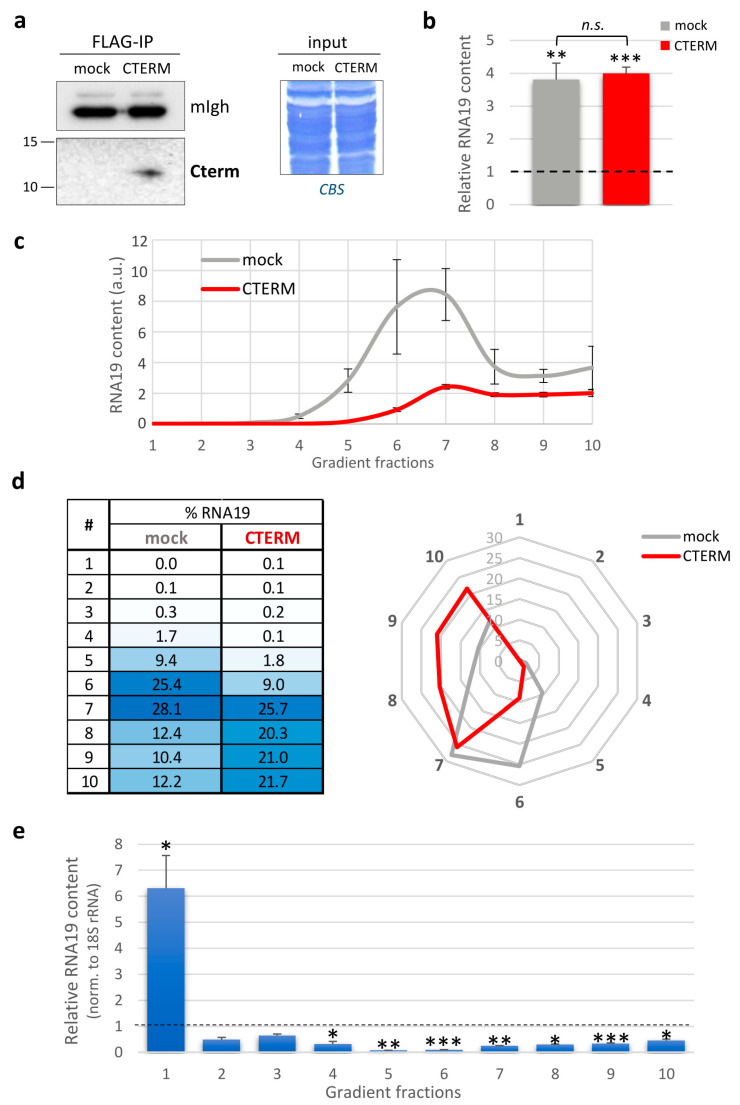
Effects of Cterm expression on RNA19 in MELAS cybrids. (**a**) Western blots of mitochondrial soluble fractions (600 µg) immunoprecipitated via anti-FLAG antibody from mock and Cterm-expressing MELAS cells were probed with antibodies against the FLAG epitope (left panel). Heavy chain of mouse IgG (mIgh) from secondary antibody was used as loading and transfer control. Before anti-FLAG pull-down, equivalent amounts of mitochondrial proteins (input, right panel) were confirmed using Coomassie blue staining (CBS). (**b**) Relative quantification of RNA19 steady-state levels using RT-qPCR. The relative RNA19 content in cybrids transfected with the empty vector (mock, grey) and Cterm-expressing MELAS (CTERM, red) was compared to wild-type cybrids (dotted line, fixed as 1-value), normalised to 18S rRNA (reference transcript). Results are presented as the mean ± SE (*n* = 4). Statistical analysis was performed using the two-tailed Student’s *t* test (n.s., not significant; **, *p* < 0.01; ***, *p* < 0.001). (**c**) The RNA19 abundance in sucrose gradient fractions from mock cybrids (grey) and MELAS expressing Cterm peptide (red) was determined using RT-qPCR analysis. Data are expressed as arbitrary units, and representative curves are presented as the mean ± SE (*n* = 4). (**d**) The distribution of RNA19 in the different fractions was determined as described for Figure 2c. Data are graphically represented by a heat table (left) and a radar chart (right). (**e**) Bars represent the relative RNA19 content calculated as the ratio between MELAS cybrids expressing Cterm and the mock control (dotted line, fixed as 1-value), normalised to 18S rRNA. Results are presented as the mean ± SE (*n* = 4). Statistical analysis was performed using the two-tailed Student’s *t* test (*, *p* < 0.05; **, *p* < 0.01; ***, *p* < 0.001).

Firstly, Cterm expression was confirmed using RT-qPCR (Figure S4). Unlike HEK293 cells, we found that Cterm peptide levels were very low in the MELAS cybrids and, consequently, the peptide was undetectable in whole-cell lysates. For this reason, to confirm the expression at the protein level, we enriched the Cterm by immunoprecipitation via anti-FLAG antibody from the mitochondrial soluble fraction of stably transfected cybrids. Following immunoblotting, a product of the expected length (approx. 12 kDa) was detected in the MELAS cells expressing the Cterm, while no signal was observed in the mock control cells (Figure 4a).

Upon Cterm expression, we measured the steady-state level of RNA19 and made a comparison with either the wild-type cybrids or the MELAS cells transfected with the empty vector (mock). As shown in Figure 4b, we found a four-fold increase in the MELAS cybrids overexpressing the Cterm compared to the wild-type cells, but no significant changes were observed when compared to the mock control.

Intriguingly, Cterm-expressing cells had a different RNA19 sedimentation profile with respect to the mock control cells (Figure 4c). Upon Cterm expression, RNA19 was distributed more evenly in the denser fractions, with a decrease in fractions 6 and 7, which typically contain the mt-LSU (Figure 4d). Most importantly, quantitative analyses performed on each fraction for RNA19 transcript, normalised to the 18S rRNA, showed a significant decrease in RNA19 in the denser fractions and an over six-fold concomitant accumulation in fraction 1, where both the free tRNA pool and the Cterm sedimented (Figure 4e). Therefore, the Cterm did not affect the total increased amount of RNA19 in the MELAS cybrids but perturbed its sedimentation profile, with a selective accumulation in the fraction where the peptide itself co-sediments. The implications of these findings, together with the evidence that the Cterm expression recovered defective mitochondrial translation in mutant cells [17], will be discussed in depth in the next section.

## 4. Discussion

### 4.1. RNA19 Sedimentation Profile in Pathological Conditions

The MELAS syndrome is a mitochondrial disease caused by point mutations localised on mtDNA genes encoding tRNA. One of the most studied mutations associated with the MELAS syndrome is the A>G transition at position 3243 of the mitochondrial mt-TL1 gene coding for tRNALeu^UUR^. Interestingly, for decades, it has been observed that patient tissues and derived cells carrying this transition or other point mutations in the same mt-tRNALeu^UUR^ increased the steady-state level of RNA19, a 2.6 kB long unprocessed mitochondrial precursor consisting of 16S mt-rRNA, mt-tRNALeu^UUR^ and mt-ND1 mRNA [6,10,13,14,15,16].

Several papers reported that the depletion or mutations in proteins involved either in the assembly of mitoribosomal subunits (PTCD1, FASTKD5) or in the processing of polycistronic transcripts (TRMT10C, MRPP2, MRPP3 and ELAC2) were also related to an increase in RNA19 [2,24,25,26,27]. In contrast, the knockdown of GRSF1, a factor that interacts with TRMT10C and regulates mt-RNA processing, caused a decrease in RNA19 steady-state levels [29]. A decrease was also observed following gene silencing of TEFM [30], an important factor acting during transcription elongation and subsequent mt-RNA processing [31]. Interestingly, the knockdown of TEFM caused a decrease in the levels of all mature transcripts and RNA19 but not all other precursor RNAs. This evidence, together with the observation that RNA19 is stable and physiologically polyadenylated with about 50 adenosines [5,14], makes this transcript more similar to a mature mRNA than to a precursor.

Furthermore, the singularity of this transcript resides in its constitution: RNA19 includes, in fact, three distinct species of RNA (rRNA, tRNA and mRNA), all structurally important for their interaction with the mitoribosome but completely different from a functional point of view. This ‘chimeric’ nature, combined with its relative abundance compared to other precursors, makes RNA19 a potentially important molecule for mitochondrial protein synthesis at both the physiological and pathological levels.

With these premises, the experimental work here reported was aimed at characterising the possible involvement of RNA19 in the pathogenesis of the MELAS syndrome. Firstly, we quantitatively confirmed the accumulation of this RNA in our MELAS cybrids compared to control cybrids. A likely explanation for this is that the mutation in mt-tRNALeu^UUR^ impairs the processing of RNA19, causing its increase. Alternatively, RNA19 accumulation could derive from a generalised increase in mitochondrial transcription as a compensatory response to either the translation deficit or the defective RNA processing [26]. Regardless of what causes RNA19 accumulation, it is intriguing that this mitochondrial RNA precursor could have a direct effect on translation, particularly on the mitoribosomes. Furthermore, it has been demonstrated that mtRNA processing is rate-limiting and required for the accurate assembly of the ribosomal subunits and the 55S monosome [26].

For this reason, we preliminarily analysed the assembly of mitoribosomal subunits in MELAS cybrids compared to controls; isokinetic sucrose gradient experiments showed that both the mt-SSU and mt-LSU were correctly assembled, although these mutant cybrids exhibited a large deficit in mitochondrial protein synthesis. Most importantly, our quantitative RNA sedimentation analyses performed on these cells showed that more than half of the total RNA19 accumulated in the gradient fractions containing the mt-LSU, pointing to an interaction between the RNA precursor and the large mitoribosomal subunit. This could not be unusual, as it was reported that ribosomal proteins can co-transcriptionally assemble on the still unprocessed 16S mt-rRNA [26,27]. Therefore, what would be the nature of the RNA19/mt-LSU association?

The answer might reside in the ‘chimeric’ feature of RNA19. One possibility is that RNA19 may be normally assembled in the mt-LSU in place of 16S mt-rRNA, affecting the mitoribosome during the elongation step of translation (‘ribosome stalling’ model, [8]). In support of this, RNA19 has been shown to bind PTCD1, a protein required for 16S mt-rRNA maturation and ribosome assembly [32]. Indeed, from the inspection of the most recent 3D structure of the human mitoribosome at 2.2 Å resolution [33], the hypothetical ‘tail’ of RNA19 (consisting of mt-tRNALeu^UUR^ and mt-ND1 mRNA) would protrude from the surface of the mt-LSU in close proximity to uL3m, bL17m and mL39 (Figure S5). The latter, together with mL45, is part of a protein layer that surrounds the polypeptide exit site; it shows homology with RNA-binding proteins whose regions for RNA binding are oriented towards the solvent and not used for interaction with rRNA [34]. Therefore, the structural arrangement of the RNA19 ‘tail’, stabilised by RNA–protein interactions with MRPs, might interfere with the tunnel exit region of the mitoribosome and the outgoing polypeptide, which in turn could lead to the stalling of the ribosome undergoing translation. From a quantitative point of view, RNA19 represents about 1% of the total 16S mt-rRNA; however, since mitochondrial mRNAs are translated on the polysomes, even a slightly increased level of incorporated RNA19 could have a global negative effect on mitochondrial translation [8].

According to an alternative model, RNA19 could be loaded onto the mitoribosome as messenger RNA, considering that it includes the mt-ND1 ORF and is normally polyadenylated [5,14]. Consistently, the mature mt-ND1 transcript normally co-sediments with the mt-LSU through the sucrose gradient [35], exactly as reported here for RNA19. It is known that most mitochondrial mRNAs are bound by mitoribosomes despite either lacking 5’ UTRs and Shine–Dalgarno-like sequences or carrying very short 5′UTRs [36,37]. Therefore, it is theoretically possible that RNA19 is approached by the mt-SSU but not the 55S assembled monosome to participate in translation initiation [38,39]. This scenario would fit the idea that, in MELAS cybrids, it is the initiation rate of mitochondrial translation that is slowed down and not the elongation step. This hypothesis was based on the observation that cells carrying the 3243A>G mutation showed decreased mt-mRNA ribosome loading, with the preferential formation of monosomes and disomes over polysomes [12].

### 4.2. The Rescuing Activity of the Isolated LARS2 C-Terminal Domain

From a therapeutic perspective, there is currently no cure for MELAS or most mitochondrial diseases, but only treatments focused on managing symptoms and preventing stroke-like episodes [40]. Among the various proposed molecular therapies, the administration of small peptides has been shown to be promising for the improvement in the MELAS clinical phenotype [41]. In particular, it has been demonstrated that overexpression of the human mitochondrial leucyl-tRNA synthetase (LARS2) carboxy-terminal domain, shortly named Cterm, proved effective in rescuing the MELAS phenotype associated with the 3243A>G transition in mt-tRNALeu^UUR^ [18,19]. Recently, our group has shown that Cterm overexpression rescued the defective mitochondrial protein synthesis in MELAS cybrids and that the peptide was able to bind RNA19 in cellulo [17]. Here, we have shown that the exogenously expressed LARS2 C-terminal peptide did not associate with the mitoribosomal subunits but sedimented in the top gradient fraction, which mainly consists of pools of tRNA and proteins that are not involved in the formation of larger protein complexes. Remarkably, Cterm-expressing MELAS cybrids showed a different RNA19 sedimentation profile compared to that of the control cybrids. In particular, RNA19 shifted from the sucrose gradient denser fractions containing the mt-LSU to the top fraction, where the Cterm sedimented. It should be emphasised that Cterm expression did not, however, decrease the steady-state levels of RNA19, indicating that the presence of the peptide did not improve the defective processing of the RNA precursor, which eventually caused its accumulation in the MELAS cybrids.

Our data are consistent with a model whereby, in cybrids carrying the 3243A>G transition (and possibly in other mutant cell lines), the Cterm peptide would be able to sequester the excess of RNA19, avoiding its accumulation on the mitoribosomes and improving the capacity of the translation machinery to synthesise mitochondrial polypeptides. This quantitative enhancement of translation does not necessarily correspond to a qualitative improvement in de novo synthesised polypeptides, bearing in mind that the pool of mutated mt-tRNALeu^UUR^, defective in aminoacylation [42] and lacking the wobble base modifications [43], would induce the incorporation of the wrong amino acids. Finally, it cannot be ruled out that RNA19 also associates with large extra protein complexes localised to mitochondrial RNA granules (mt-RNA processing machinery, MRG-clustered mitochondrial aa-tRNA synthetase [44] or others).

## 5. Conclusions

The reported results did not allow us to discriminate whether RNA19 is incorporated into the mitoribosome, loaded as mt-mRNA, or interacts with mt-LSU proteins on the ribosome surface. However, the circumstance that, in the presence of the Cterm, RNA19 shifted from the denser to the top fraction of the gradient would point to RNA19 not being embedded into the mitoribosome. Nevertheless, it is still possible that a small fraction of the precursor assembles with the MRPs to form the mt-LSU; this could be a way to physiologically regulate the speed of the polysomes proceeding along the mRNA during the polypeptide elongation step. Evidently, under pathological conditions as observed in the MELAS cybrids, the accumulation of RNA19 could somehow stall the mitoribosomes and either delay or block the translation process. In addition, mt-tRNALeu^UUR^ and, by extension, RNA19 may have some other yet-unknown functions in mitochondrial translation. Indeed, it is known that tRNAs can play roles other than the canonical one of carrying amino acids to the ribosome [45]. An important example is given by the unexpected discovery that, in human mitoribosomes, mt-tRNAVal is normally embedded into the mt-LSU in place of 5S rRNA, acquiring an additional structural function [46,47]; in porcine mitoribosomes, a different tRNA, mt-tRNAPhe, has been found. Intriguingly, depending on the organisms and tissues considered as well as the cellular adaptive conditions, different species of mitochondrial tRNAs can be structurally integrated into the mitoribosome [48].

In conclusion, we suggest RNA19 as a potential regulatory molecule in both physiological and pathological conditions, mediating the beneficial effect of the isolated LARS2 C-terminal domain on mitochondrial translation. This would be yet another example of a mitochondria-derived RNA that plays an important role in mitochondrial metabolism, contributing to the regulation of cell physiology [49]. In view of therapeutic strategy developments, further experimental work will be needed to deepen our knowledge of the molecular interactions between the Cterm peptide, RNA19 and the mitoribosomes and shed more light on their functional relevance in pathological conditions.

## Data Availability

Not applicable.

## Supplementary Materials

The following supporting information can be downloaded at: https://www.mdpi.com/article/10.3390/life13091863/s1. Figure S1: RFLP analysis and quantification of heteroplasmy in control and MELAS cybrids; Figure S2: RNA19 abundance in sucrose gradient fractions from HEK293 cells; Figure S3: Pattern of RNA extracted from 10–30% sucrose gradient fractions; Figure S4: Analysis of Cterm transcript using RT-qPCR; Figure S5: Structure of human mitoribosome at 2.2 Å resolution.
