# Supplementary material for: Molecular Investigation of Mitochondrial RNA19 Role in the Pathogenesis of MELAS Disease
Source: Life (Basel). 2023 Sep 3;13(9):1863. doi: 10.3390/life13091863 (PMC10532844; doi:10.3390/life13091863)
Supplement: Supplementary file 1 [file life-13-01863-s001.zip › life-2541199-supplementary.pdf]

Supplementary Materials

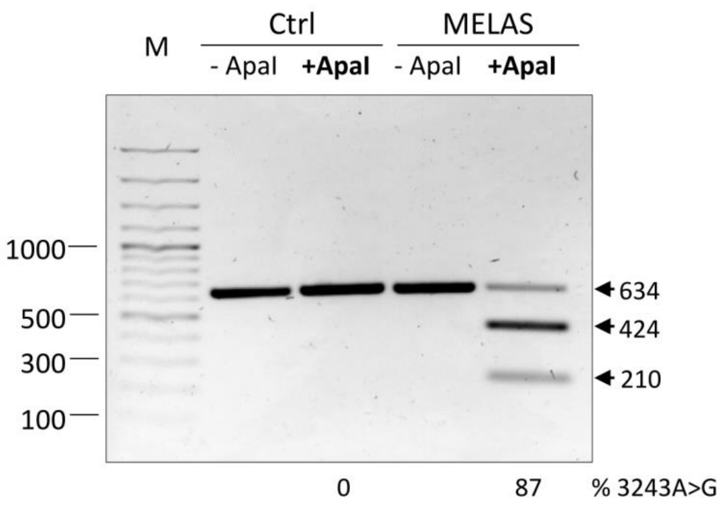

Figure S1. RFLP analysis and quantification of heteroplasmy in control (Ctrl) and MELAS cybrids. Digestion products by ApaI were separated onto a 1.5% agarose gel. Mutation load percentage (bottom) was determined as proportion of mutant (424 bp and 210 bp products) to wild type (634 bp product) mtDNA. Band intensities were quantified by ImageQuant TL 8.1 software (GE Healthcare Life Sciences).

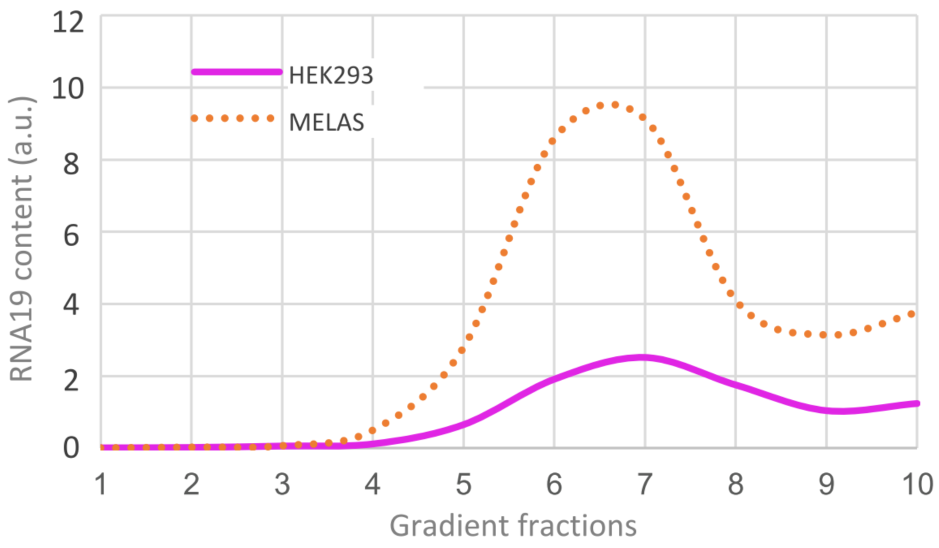

Figure S2. RNA19 abundance in sucrose gradient fractions from HEK293 cells. The precursor transcript content in each fraction from HEK293 cells (purple line) and MELAS cybrids (orange dotted line) was determined by RT-qPCR analysis, as detailed in Methods. Data are expressed as arbitrary units.

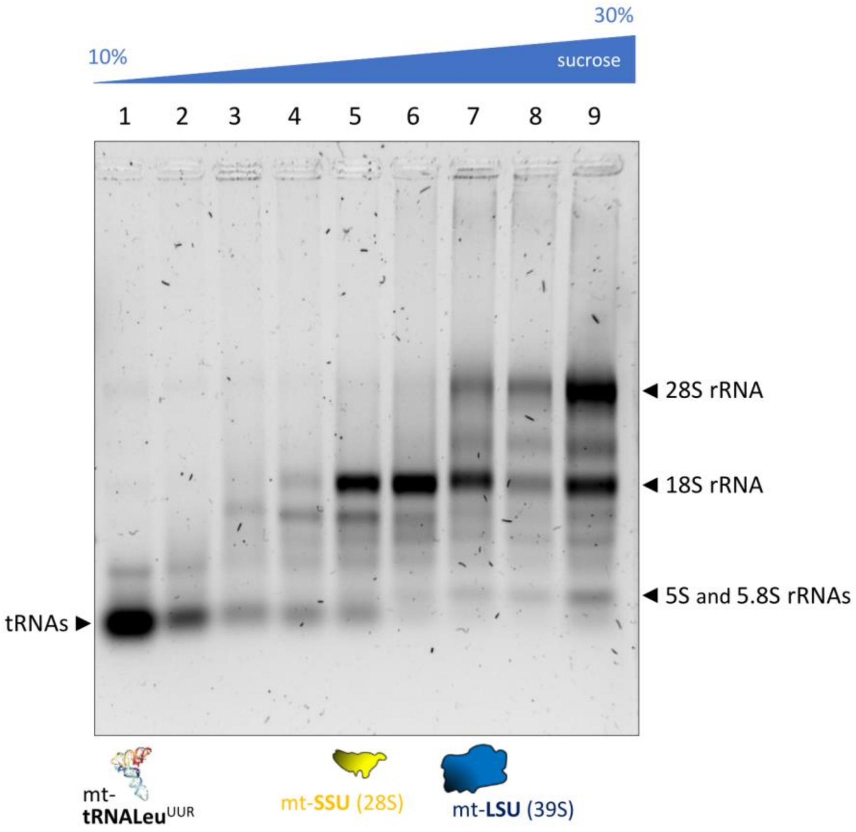

Figure S3. Pattern of RNA extracted from 10%-30% sucrose gradient fractions. RNA species were separated onto 1.5% agarose gel and stained with SYBR™ Gold (ThermoFisher Scientific, Carlsbad, CA, USA). Most abundant RNAs are indicated on both sides.

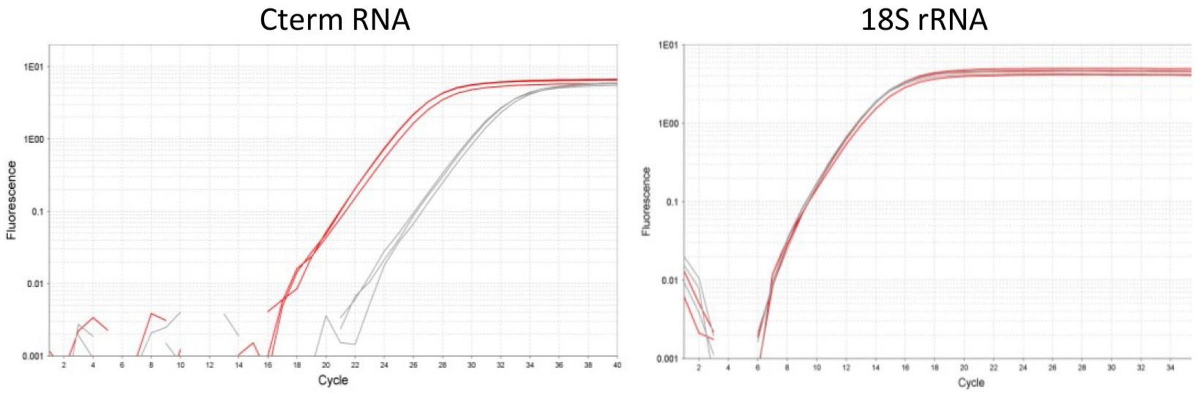

Figure S4. Analysis of Cterm transcript using RT-qPCR. Left panel: the expression of Cterm-FLAG RNA was assessed in MELAS cybrids transiently transfected with either empty pcDNA5/FRT/TO® vector (mock, grey curves) or MTS-Cterm-FLAG vector (CTERM, red curves). Amplification reactions were carried out by using the forward primer designed on Cterm ORF and the reverse primer designed on FLAG sequence (as detailed in Section 2.6). Right panel: nucleus-encoded 18S rRNA was used as endogenous control.

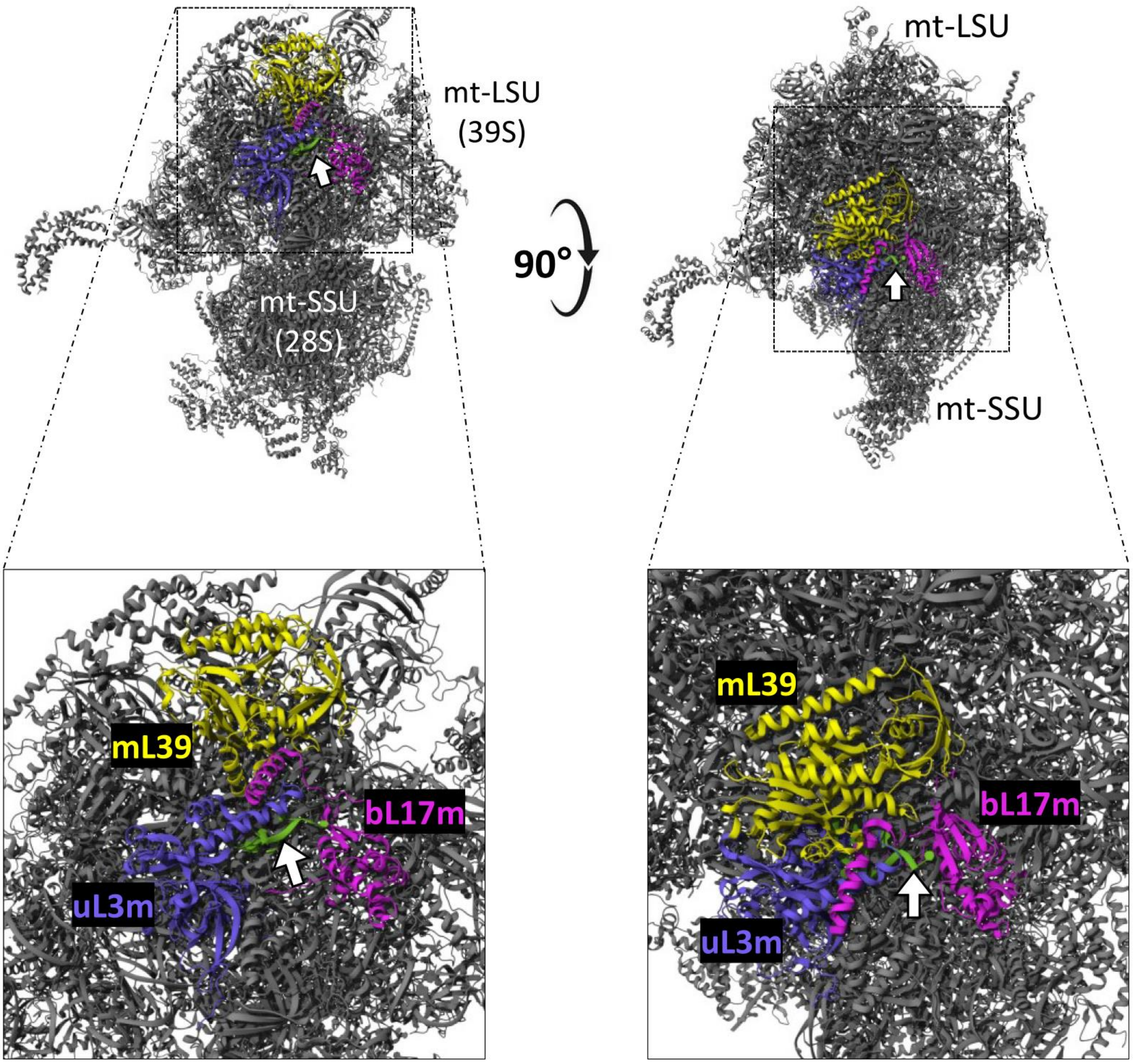

Figure S5. Structure of human mitoribosome at 2.2 Å resolution. Bottom insets zoom on the region locating the 16S mt-rRNA 3′ end (green, pointed by a white arrow), where the RNA19 'tail' might protrude externally to the mt-LSU. Surrounding MRPs (mL39, yellow; uL3m, blue; bL17m, purple) are depicted. Images were generated from PDB ID: 7QI4 using the *3D View* tool available at the RCSB PDB website (http://www.rcsb.org/).
